# Prevention and control of cardiovascular disease in “real-world” settings: sustainable implementation of effective policies

**DOI:** 10.3389/fcvm.2024.1380809

**Published:** 2024-11-19

**Authors:** Shanthi Mendis, Ian Graham

**Affiliations:** ^1^ Global Health, The Geneva Learning Foundation, Geneva, Switzerland; ^2^ Cardiovascular Medicine, Trinity College, Dublin, Ireland

**Keywords:** cardiovascular disease, inequities, low- and middle-income countries, health spending, health workforce availability

## Abstract

Global progress in addressing cardiovascular diseases (CVD) has been insufficient to attain the nine WHO non-communicable disease (NCD) targets and the Sustainable Development Goal (SDG) target of reducing premature NCD mortality by one-third by 2030. Progress has been slowest in low- and middle-income countries (LMIC) where addressing the CVD burden is a foremost development imperative. This review examines the reasons for this situation to propose a way forward. First, we review policy instruments to address behavioral and metabolic risk factors of CVD and health system interventions to improve cardiovascular outcomes. Second, we illustrate the financial, health workforce, health system challenges, and weak national capacity that impede the implementation of these policy instruments. Third, we discuss how LMIC might move forward despite these challenges by (a) giving due consideration to contextual and other factors that determine the success of policy implementation (b) including affordable, high-impact interventions as the core of the universal health coverage health benefit package with primary health care as the foundation and (c) by taking note of the WHO guidance provided in the 2023–2030 implementation roadmap for the Global Action Plan for prevention and control of NCD.

## Introduction

The impact of non-communicable diseases (NCD), including cardiovascular diseases (CVD), increased from causing 61% of global deaths in 2000 to 74% (or 41 million) of global deaths in 2019 and from causing 47% of global disability-adjusted life years (DALYs) in 2000 to causing 63% of DALYs in 2019 (1). In 2019, CVD, diabetes, cancer, and chronic respiratory disease were collectively responsible for 81% of NCD deaths. The highest number of NCD deaths was from CVD with 17.9 million deaths (1).

CVD are increasingly significant causes of premature morbidity and mortality, particularly in low- and middle-income countries (LMIC) (2, 3). They disproportionately affect individuals in lower socioeconomic classes (4). Worldwide, CVD impart a heavy economic burden on healthcare systems (5). Furthermore, ill health and premature mortality due to CVD reduce labor productivity and economic growth (6). In addition, seeking care for CVD in LMIC often results in high out-of-pocket spending by households and worsening of poverty (7).

LMIC have to grapple with the rising burden of NCD, including CVD, together with other competing health priorities such as mental health, maternal and child health, and communicable diseases. Consequences of climate change, conflicts, and humanitarian crises add to the workload. However, despite all these challenges, even low-income countries must accelerate national responses to address the CVD burden by adopting incremental and pragmatic approaches before it completely overshadows their growth and development prospects.

## Methodology

Numerous peer-reviewed publications have summarized the scope and implementation challenges of population-based and individual-level cardiovascular policies. A knowledge synthesis was done of a body of literature which describes the current status of prevention and control of CVD while providing new insights on advancing the field of cardiovascular policy and program implementation. The purpose was not to provide a comprehensive review of the literature but to present a contextualized overview of broad CVD policy and program areas that are scalable and sustainable in resource-constrained settings. The article is a condensation of the current World Health Organization (WHO), World Bank, and United Nations (UN) reports (*n* = 49) and guidelines (*n* = 5) on this subject supported by relevant literature obtained from a Medline search. It includes publications pertaining to evidence synthesis (40 systematic and scoping reviews), implementation of cardiovascular policies, and programs with a special focus on LMIC (*n* = 29) and epidemiology of CVD (*n* = 17).

## Policy instruments to address behavioral and metabolic risk factors for CVD

The substantial decline in CVD mortality in high-income countries over the last four decades has been attributed, among other things, to the success of public health policies to reduce population exposure to cardiovascular risk factors and health system strategies that improved medical care through early diagnosis and treatment (8–10).

Compelling evidence demonstrates that tobacco use, harmful use of alcohol, an unhealthy diet, physical inactivity, and air pollution are risk factors for CVD and their metabolic biomarkers (e.g., diabetes, hypertension, and hyperlipidemia) (11–18). A substantial body of evidence, mostly from developed countries, also shows that tobacco use, harmful use of alcohol, unhealthy diet, physical inactivity, and metabolic biomarkers of CVD are modifiable with appropriate policies and legislative and regulatory measures (19–36).

For example, various educational policies, including awareness programs, health warnings on tobacco and alcohol products, and food nutrition labeling, have been widely used to reduce the consumption of unhealthy commodities (19–24). Regulatory policies such as smoking bans in public places and restricting the availability of unhealthy products have also given positive results (19–22, 25–28). Furthermore, fiscal policies, in the form of higher prices and taxes on tobacco, alcohol, sugar-sweetened beverages (SSB), and junk foods, have been shown to promote healthy behaviors and positively impact health outcomes (19–22, 29–34). Notably, estimates suggest that if revenues from health taxes on tobacco, alcohol, and unhealthy food were invested in health systems, these could significantly close the revenue gap of LMIC in the short term (35). A recent systematic review of 33 studies indicates that population-level interventions to promote physical activity may effectively prevent CVD and diabetes when implemented with due consideration of local contextual factors (36).

## Health system policies and interventions

There is a range of evidence-based health system interventions for preventing and treating CVD (37–63). They address cardiovascular risk (38–45), cardiovascular disease in ambulatory settings (46), acute coronary syndromes and stroke (47–52), heart failure (53, 54), prevention of rheumatic heart disease (55, 56), atrial fibrillation (57), revascularization interventions (58, 59), and rehabilitation of CVD (60). If primary health care (PHC) is used as a foundation, some of these interventions can be implemented very cost-effectively even in LMIC as core components of a universal health coverage (UHC) policy (61–63). An example is early detection and treatment of behavioral and metabolic risk factors of CVD in primary care using a total risk approach (20). In resource-constrained settings, diverting a high proportion of resources to less cost-effective high technology tertiary care interventions will tend to widen health inequities (6, 19, 20).

## Inadequate national capacities for implementation of cardiovascular policies

National capacity is critical for the successful implementation of CVD policies and programs. The WHO has monitored national capacities periodically since 2001 using country capacity surveys. In the survey completed by the Ministry of Health, countries are requested to provide information on the following items relating to NCD: (i) public health infrastructure, partnerships, and multisectoral collaboration; (ii) policies, strategies, and action plans; (iii) health information systems and surveillance; and (iv) health system capacity for detection and treatment. All WHO Member States (*n* = 194) responded to the most recent survey conducted in 2021 (64). Although most countries (86%) have included NCD in their national health plans, only two-thirds (65%) had set NCD targets aligned with the nine voluntary global targets of the global NCD action plan (65) (Table 1). A national multisectoral mechanism to oversee policy coherence and accountability of sectors beyond health was present in only 46% of countries. Over half of countries (55%) reported that their policies were multisectoral and covered all four behavioral risk factors and all four main NCD, including CVD.

**Table 1:** Nine voluntary global NCD targets to be attained by 2030 (66).

1.	A one-third relative reduction in the overall mortality from cardiovascular diseases, cancer, diabetes, or chronic respiratory diseases.
2.	A 20% relative reduction in the harmful use of alcohol.
3.	A 15% relative reduction in the prevalence of insufficient physical activity.
4.	A 30% relative reduction in the mean population intake of salt/sodium.
5.	A 30% relative reduction in the prevalence of current tobacco use.
6.	A 25% relative reduction in the prevalence of raised blood pressure or to contain the prevalence of raised blood pressure.
7.	Halt the rise in diabetes and obesity.
8.	At least 50% of eligible people (aged 40 years and older with a 10-year cardiovascular risk ≥20%) including those with CVD to receive drug therapy and counseling (including glycemic control) to prevent heart attacks and strokes.
9.	An 80% availability of the affordable basic technologies and essential medicines, including generics, required to treat major non-communicable diseases in both public and private facilities.

Taxation on alcohol and tobacco was implemented by many countries (97% and 88%, respectively). Other fiscal measures, such as taxation on sugar-sweetened beverages (47% of countries) and food high in fats, sugar, or salt (13% of countries), remained underutilized. A third of countries had policies to reduce the intake of saturated fat (35%) or to eliminate trans fat (34%), and 53% of countries reported having a salt reduction policy in place. Only around a third of countries (38%) were implementing policies to reduce the impact of the marketing of unhealthy foods on children. Policies to promote physical activity were implemented by under half of countries. Overall, the four WHO country capacity surveys indicate that an increasing number of countries are committed to developing policies and plans to address NCD. However, the resources and structures needed for effective implementation long-term need to be improved in most LMIC (67).

## Insufficient global progress despite evidence-based policies and interventions

Since 2010, the progress made in NCD prevention and control, as well as barriers to progress, has been captured in 11 reports by the WHO Director-General (68–78) and four reports by the Secretary-General of the United Nations (UN) (79–82). The Heads of State have made 66 commitments at three high-level meetings of the UN General Assembly in 2011, 2014, and 2018 to accelerate the global and national NCD responses (83–85). In addition, in 2019, at the UN high-level meeting on UHC, a commitment was also made to strengthen further efforts to address NCD, including CVD (86).

Despite an array of policies, interventions, and high-level commitments, the progress made by countries has been insufficient to attain the nine voluntary targets of the Global NCD Action Plan (65) and the Sustainable Development Goal (SDG) target of 3.4 (30% reduction of premature NCD deaths by 2030) (87, 88). Only 35 countries (for women) and 30 countries (for men) are on track to meet SDG target 3.4. Most of these are high-income countries. There has also not been a significant improvement in the global trends of CVD risk factors, except for tobacco, over the past decade (89). Furthermore, despite substantial advancements in our understanding of disease progression, heart attacks and strokes are increasingly diagnosed in younger age groups (90, 91), a distressing trend. It signals increased exposure of the younger generation to behavioral risk factors such as poor diet, tobacco and alcohol use, and physical inactivity.

## Grave resource inequalities between countries

There appears to be an insufficient acknowledgement of the complexity of sustainable implementation of policies and programs in “real-world” settings in LMIC. Any initiative to accelerate prevention and control of CVD in LMIC must factor in resource inequalities between countries. In addition, deficiencies across all health system building blocks, including health financing, health workforce, governance, health information, access to supplies and medicines, referral pathways, and service delivery (19, 20), need to be given due consideration.

Only about 14% of those who die of NCD before the age of 70 years are from high-income countries. However, there is a stark contrast in the distribution of global health spending compared to needs. In 2022, high-income countries with 15% of the world's population accounted for about 80% of total global health spending of US$ 9.0 trillion (92). Upper-middle-income countries, with 33% of the world population, accounted for 16%; lower-middle-income countries, with 43% of the world's population, for just under 4%; and low-income countries, with 8% of the world population for approximately 0.2%.

Furthermore, about 11% of the world's population lives in countries that spend less than US$ 50 per person per year. Per capita health spending was US$ 39 in low-income countries, US$ 125 in lower-middle-income countries, US$ 515 in upper-middle-income countries, and US$ 3,708 in high-income countries. Health ﬁnancing trends and worsening macro-fiscal conditions suggest that health spending gaps between low-income and high-income countries are unlikely to narrow in the next decade (93).

As of 2021, about half the world's population—4.5 billion people—was not covered by essential health services, and about two billion people experienced financial hardship due to out-of-pocket health spending, including 344 million people living in extreme poverty (94). Higher government spending on health in LMIC is essential to ensuring equitable access to health services and easing household financial hardship.

Globally, the needs-based shortage of healthcare workers in 2013 is estimated to be about 17.4 million, of which almost 2.6 million are doctors, nearly 9 million are nurses and midwives, and the remaining include other health worker cadres. The most significant shortages of health workers are in Southeast Asia, at 6.9 million, and Africa, at 4.2 million. The global needs-based shortage of healthcare workers is projected to decline only by about 17% by 2030 (95) (Table 2).

**Table 2:** Total numbers of health workers needed to reach the SDG threshold estimated for 2013 and forecasted for 2030 (by income group) (95).

Income	Physicians		Nurses/midwives		Other cadres		Total health workers		% Change
	2013	2030	2013	2030	2013	2030	2013	2030	
High	1,612,259	1,704,610	4,058,748	4,291,235	2,115,214	2,236,375	7,786,221	8,232,220	6%
Upper middle	3,069,815	3,354,235	7,728,044	8,444,051	4,386,610	4,793,032	1,518,4469	1,659,1318	9%
Lower middle	3,274,396	4,088,220	8,243,062	1,029,1805	6,323,837	7,895,573	1,784,1295	2,227,5598	25%
Low	991,190	1,403,036	2,495,252	3,532,045	2,075,236	2,937,510	5,561,678	7,872,591	42%

Given the above resource constraints, health system policies and programs that have been effective in high-income countries, such as single-risk factor programs on hypertension or diabetes, are unlikely to be scalable or sustainable in most LMIC. Single-risk factor hypertension control programs are resource-intensive, given that hypertension has a high prevalence in adults (20%–25%) and a very high prevalence in those above 60 years (higher than 50%). However, it is imperative that in these settings, all cardiovascular risk factors—hypertension, diabetes, hypercholesterolemia, and tobacco use—be addressed. This is feasible, provided hypertension, diabetes, and hyperlipidemia are addressed collectively in PHC using a more cost-effective total cardiovascular risk approach (61, 62, 96). This approach enables available resources to be targeted at those who are most vulnerable to developing heart attacks and strokes: individuals with established CVD or with moderate–high cardiovascular risk (20). This approach is particularly well-suited for LMIC (Table 3), and there are many successful examples of its implementation (20).

**Table 3:** Advantages of the total risk approach compared to the single-risk factor approach.

	Total risk approach	Single-risk factor approach
1	Recognizes that the risk of developing a heart attack or a stroke is determined by the combined effect of a number of risk factors (age, gender, tobacco use, blood pressure, blood cholesterol, and diabetes).	The main focus is on hypertension defined by using an arbitrary cutoff of 140/90 mmHg.
2	A very cost-effective intervention which can be scaled-up in primary care even in resource-constrained settings.	Less cost-effective intervention which may not be affordable and sustainable to low-income countries.
3	Enables the use of limited resources on those at moderate to high risk to prevent heart attacks, strokes, and renal impairment.	Management decisions based on blood pressure thresholds alone may result in people with blood pressure between 140/90 and 159/99 mmHg without any other risk factors (about 60% of the hypertensive population) being started on lifelong medications despite a low overall cardiovascular risk.
4	Numbers requiring pharmacological treatment can be adjusted to match resource availability.	Drug treatment instead of non-pharmacological treatment of people with BP 140/90 to 159/99 mmHg without any other cardiovascular risk factors may not be affordable in low resource settings as prevalence of hypertension is high (20 to 25%).
5	Total risk assessment requires information on smoking, diabetes, hyperlipidemia, and obesity.	Hypertension may be treated without due regard to other comorbidities and risk factors which impact on outcomes.
6	Facilitates integration and improves efficiency of primary care programs because hypertension, diabetes, hyperlipidemia, smoking, and obesity can be managed in one clinic.	Less efficient primary care program because different guidelines and clinics are required to manage different risk factors.
7	Facilitates detection and management of diabetes, hyperlipidemia, smoking, and other risk factors.	Other risk factors such as diabetes, smoking, and hyperlipidemia may be missed in busy clinics.
8	Risk prediction color charts are useful for educating and motivating people to adopt healthy behaviors.	People find it difficult to interpret blood pressure numbers are levels.
9	Misdiagnosis and overtreatment of hypertension unlikely.	Misdiagnosis and overtreatment of hypertension more likely.
10	Follow-up visits and referral to higher levels of care can be tailored to the level of cardiovascular risk.	Follow-up visits and referral to higher levels of care, guided by blood pressure thresholds.

## Key considerations for effective implementation of policies

Inadequate consideration of critical factors determining the successful implementation of policies and programs in “real-world” settings is an essential reason for slow progress in attaining global NCD targets (97). These factors include (i) context, (ii) sustainability, (iii) timeline, and (iv) unintended consequences.

Contextual factors, including intervention costs, feasibility, and scalability in specific settings, are essential for successful implementation. For example, healthcare budgets and organizational support (e.g., availability of health workers, staff workload, and primary care capacity) in LMIC are different from those in high-income countries. Political leadership, public health capacity, and multisectoral collaboration, identified as factors supporting public policy implementation, are also far from robust in most LMIC (98).

Sustainability is another critical consideration. For example, prioritizing only emergency care of myocardial infarction and stroke over implementation of WHO Best Buy policies and interventions is unlikely to be a sustainable approach to address the CVD burden in any LMIC. Sustainability could be strengthened by innovative financing strategies, such as taxation on unhealthy products (e.g., tobacco, alcohol, and sugary drinks) to fund NCD programs.

Vertical NCD programs initiated with short-term external aid may not be viable unless domestic funds are available for long-term implementation. Even public health policies (e.g., policies to control tobacco use, alcohol, unhealthy diet, physical inactivity, and air pollution) and PHC programs (screening, treatment, and follow-up) will need to be sustained over a long period of time to exert an impact. This requires uninterrupted resource allocation and attention to dynamic processes involved in successful implementation. Furthermore, unstable political contexts in most LMIC often make maintenance of implementation processes over time quite challenging (98, 99).

Another major challenge of policy and program implementation is the timeline. Adopting and implementing policies and programs must align with a favorable window of opportunity in the policy environment. For example, currently, as part of the global movement on UHC, many LMIC are legitimizing action for health system reforms. This is an opportunity to incorporate a core set of very cost-effective and high-impact CVD policies and interventions (e.g., WHO Best Buys, Table 4) in a health benefit package.

**Table 4:** WHO Best Buys for prevention and control of cardiovascular diseases (100, 101).

Risk factor/disease to be addressed	Interventions	Detailed description
Reduce tobacco use	Taxation Packaging Advertising, promotion, and sponsorship Smoke-free public policies Health education	Increase excise taxes and prices on tobacco products. Implement plain/standardized packaging and/or large graphic health warnings on all tobacco packages. Enact and enforce comprehensive bans on tobacco advertising, promotion, and sponsorship Eliminate exposure to secondhand tobacco smoke in all indoor workplaces, public places, and public transport. Implement effective mass media campaigns that educate the public about the harms of smoking/tobacco use and secondhand smoke.
Reduce harmful use of alcohol	Taxation Advertising Availability	Increase excise taxes on alcoholic beverages. Enact and enforce bans or comprehensive restrictions on exposure to alcohol advertising (across multiple types of media). Enact and enforce restrictions on the physical availability of retailed alcohol (via reduced hours of sale).
Reduce unhealthy diet	Reformulate food Supportive environment Health education Packaging	Reduce salt intake through the reformulation of food products to contain less salt and the setting of target levels for the amount of salt in foods and meals. Reduce salt intake through the implementation of front-of-pack labeling. Reduce salt intake through the establishment of a supportive environment in public institutions such as hospitals, schools, workplaces, and nursing homes, to enable lower sodium options to be provided. Reduce salt intake through a behavior change communication and mass media campaign. Reduce salt intake through the implementation of front-of-pack labeling.
Reduce physical inactivity	Health education	Implement community-wide public education and awareness campaigns for physical activity which includes a mass media campaign combined with other community-based educational, motivational, and environmental programs aimed at supporting behavioral change of physical activity levels.
Manage diabetes and cardiovascular disease including hypertension	Drug therapy and counseling	Counseling and drug therapy based on high cardiovascular risk (glycemic control for diabetes mellitus and control of hypertension using a total risk approach including individuals who have had a heart attack or stroke).

Unintended consequences of policies and programs should not be disregarded. In resource-constrained healthcare settings, where there is unmet demand, choosing to treat or care for one patient means a lost opportunity to treat or care for another patient (opportunity costs). For example, vertical single-cardiovascular risk factor programs, which are resource-intensive, can result in opportunity costs for maternal and child health programs. Furthermore, combining maternal and child health programs with NCD screening and treatment could mitigate the trade-offs between vertical and horizontal programs.

Paying attention to the unintended consequences of treatment policies is also essential. For instance, new hypertension treatment guidelines recommend a single-pill combination as the initial treatment for hypertension. Most single-pill combinations contain an angiotensin-converting enzyme inhibitor or an angiotensin receptor blocker, which should not be prescribed to women of childbearing age. Although the risk may be negligible in high-income country settings, in LMIC with limited access to antenatal health services, failure to discontinue these medications in pregnant women could cause foetal malformations (102), an eminently preventable risk. This example also highlights the importance of gender-sensitive health policies and the need of their adaptation to the specific needs and realities of women, particularly in limited resource settings.

Finally, before investing in implementation, in addition to the above country-specific factors, the Ministries of Health need to compare the cost-effectiveness, operational, and technical feasibility of different policies and programs to determine priorities. Through such priority setting, limited resources can be utilized to maximize coverage and impact (103–105). Many resources and tools including the WHO-CHOICE tool are available to guide this process (105–107).

## Limitations

This review is based on an analysis of the current literature pertaining to the prevention and control of cardiovascular disease in “real-world” settings. It is of necessity a qualitative review because there are no relevant randomized control trials in this field, particularly in regard to the implementation of effective policies. A meta-analysis of the relevant literature would be desirable, but the heterogeneity of the sources would make this methodologically problematic and open to criticism.

## Discussion: way forward

As underscored in objective one of the global NCD Action Plan, there is a continuing need to create awareness of the importance of tackling CVD, focusing more on cost-effective prevention and control policies and their roll-out (65). There are no quick fixes for complex implementation challenges which impede policy implementation for CVD prevention and control, particularly in LMIC. However, there are sustainable policy options appropriate for resource-constrained settings. The most promising pathway forward is embracing CVD policies and programs in progressive realization of UHC with PHC as the foundation. Advancing the service coverage dimension of UHC requires incremental expansion of essential health services to address CVD. Rapid scale-up of service coverage to comprehensively address CVD is unlikely to be feasible in LMIC, even with health financing reforms. A pragmatic approach would be to provide stepwise population coverage and full prepayment for a narrower scope of high-impact policies and interventions with a good return on investment, such as the WHO Best Buys (101, 108).

WHO Best Buys (Table 4) include 13 very cost-effective policy interventions to control tobacco and alcohol use, unhealthy diet, and physical inactivity. Population level services rely on strengthening multisectoral action as well as the health system. They can achieve significant health benefits and can be rapidly implemented at low cost, provided there is expertise and capacity in the Ministry of Health.

The WHO Best Buy portfolio's most cost-effective health system intervention is the total risk approach to address cardiovascular risk factors in individuals without and with CVD (primary and secondary prevention, respectively. Updated WHO risk charts, integrated protocols, and training material are available to deliver this PHC intervention even by non-physician health workers (62, 109). The protocols cover metabolic risk factors (hypertension, diabetes, hyperlipidemia) and behavioral risk factors (tobacco use, harmful use of alcohol, unhealthy diet, and physical inactivity) in individuals. This intervention is captured in global NCD targets 8 and 9 and is being implemented in many resource-constrained settings (110–127).

The global NCD target 9 is on the minimum requirements for implementing this intervention: basic diagnostics (blood pressure measurement device, a weighing scale, height measuring equipment, blood sugar and blood cholesterol measurement devices with strips, and urine strips for albumin assay) and essential medicines (aspirin, statin, angiotensin-converting enzyme inhibitor, thiazide diuretic, long-acting calcium-channel blocker, beta-blocker, metformin, and insulin).

LMIC need to leverage international development aid and innovative financing models to identify adequate resources for population coverage of this essential health service to maximize the impact and efficiency of health spending. Funds must be rationally allocated to primary care to ensure the availability of at least six basic diagnostics, eight essential medicines, and an adequately trained workforce. Even low-income countries could consider the best buys as a starting point in deliberations on a new health benefits package or refinement of an existing package.

Governments particularly in LMICs need to play a more proactive role in using fiscal and pricing policies, including taxes and subsidies to promote healthy diets and for tobacco control. Currently, 45 countries including 3 middle-income countries (Barbados, Mexico, and South Africa) have introduced SSB taxes. Implementing SSB taxes have resulted in higher SSB prices, a significant reduction in sales (15%) and an estimated decline in demand (18%) (128). While taxing non-essential energy-dense food in Mexico has resulted in increased prices and reduced sales of taxed products, fruit and vegetable subsidies are associated with a moderate increase in fruit and vegetable sales (129). Further research is required to understand the implications of food taxes and subsidies for population-level consumption, diet, and health outcomes. There is strong evidence that increased taxes that are passed on to tobacco users as higher prices reduce tobacco consumption and the health harm it causes (130). At present, except 15 countries, all others are applying some type of cigarette excise tax. It is critical to set taxes at a high level to reduce the affordability of tobacco products and make regular adjustments to increase the tax rate so that it keeps up with inflation and income growth in a country over time (131).

Since obesity is a major risk factor of cardiovascular disease and type 2 diabetes, it will not be possible to advance prevention and control of CVD even in LMIC, without tackling the growing burden of obesity simultaneously. At the 75th World Health Assembly in 2022, Member States adopted new recommendations for the prevention and management of obesity and endorsed the WHO Acceleration Plan to Stop Obesity (132). All countries need to implement multi-sector country-level action recommended in this plan.

When addressing policy gaps and system bottlenecks, the Ministries of Health in LMIC could benefit from lessons learned in other countries in implementing strategies to improve healthcare worker retention (133), governance models to improve health system efficiency and accountability (134), and programs that have enhanced access to affordable essential medicines (135, 136).

It should be underscored that while this paper focuses mainly on health system and financing issues, social determinants of health (poverty, education, urbanization, and food security) play a critical role in shaping cardiovascular outcomes. Integrating policies that address these determinants into national NCD strategies through multisectoral approaches involving the education sector, agriculture, and urban planning is critical (137). Policy action on social determinants of health as well as monitoring social determinants and health equity are essential for accelerating progress in prevention and control of CVD (138).

## Actions recommended in the implementation roadmap to accelerate progress

The midpoint evaluation of the implementation of the Global Action Plan (67) noted the relatively slow progress of countries in achieving the nine global NCD targets. Based on this finding, in 2021, the 74th World Health Assembly requested that WHO develop an implementation roadmap to support the country implementation of the *Global action plan for preventing and controlling noncommunicable diseases 2013–2030* (139). The implementation roadmap recommends that countries accelerate their national response to move into a sustainable path to attain global NCD targets and SDG target 3.4 to reduce premature mortality from NCD by one-third by 2030. Recommended actions include the following:

(1) identify the barriers and opportunities for scaling up, including through assessment of the status of domestic NCD responses against the nine global NCD targets and the SDG target on NCD;
(2) increase budgets for health and NCD prevention and control in a stepwise manner through domestic, bilateral, and multilateral channels, including from health taxes on unhealthy commodities (e.g., tobacco, alcohol, sugar-sweetened beverages, and unhealthy food);
(3) generate savings by cutting wasteful health spending on cost-ineffective interventions and identifying irrational use of high-cost items such as medicines and other health products that constitute a sizeable share of public sector budgets;
(4) reduce inequities in access to essential health services, including through health financing, PHC reforms, and basic benefit packages;
(5) strengthen health systems with a particular focus on PHC and include CVD interventions in UHC health benefit packages;
(6) prioritize and scale up the implementation of the most impactful and feasible interventions with a particular focus on WHO Best Buys;
(7) accelerate capacity for multisectoral and multistakeholder collaborations, including by engaging non-State actors, taking due consideration of their potential conflict of interest with public health goals;
(8) strengthen national monitoring and surveillance systems to ensure reliable national data on NCD risk factors, diseases, and mortality for data-driven policy actions and to strengthen accountability;
(9) promote engagement of people with lived experience of NCD to co-design, implementation, and accountability of health policy reforms;
(10) strengthen the national capacity for the governance of multistakeholder engagement, cross-sectoral collaboration, and result-oriented partnerships; and
(11) collaborate with international partners to support and strengthen research and innovation by working with academic partners and research institutions in countries.

## Conclusion

All countries must improve CVD outcomes to reduce preventable morbidity and mortality. Every country still has options for achieving the global NCD targets, which can pave the way to attain the SDG target of reducing premature mortality from NCD by one-third by 2030. However, complex implementation challenges impede the national response in LMIC, and these are unlikely to ease out in the foreseeable future. To overcome these challenges, LMIC must adopt sustainable and resource-sensitive approaches. One such path which is affordable to all LMIC is to include the fourteen very cost-effective high-impact policies and interventions (WHO Best Buys that address the CVD burden) as a starting point of UHC with PHC as the foundation. When rolling out policies and programs, LMIC needs to be aware of the determinants of effective implementation: context, sustainability, timeline, and unintended consequences to maximize success. Increasing domestic investment in health and strengthening national capacity for ensuring action across government sectors will be indispensable for accelerating progress.
